# One-Stage Immediate Alloplastic Breast Reconstruction in Large and Ptotic Breasts: An Institutional Algorithm

**DOI:** 10.3390/jcm12031170

**Published:** 2023-02-01

**Authors:** Silvia Rampazzo, Noemi Spissu, Michela Pinna, Germana A. M. Sini, Emilio Trignano, Rita Nonnis, Claudia Sanna, Manuela Rodio, Matilde Tettamanzi, Corrado Rubino

**Affiliations:** 1 Plastic Surgery Unit, University Hospital Trust of Sassari, 07100 Sassari, Italy; 2 Plastic, Reconstructive and Aesthetic Surgery Training Program, University of Sassari, 07100 Sassari, Italy; 3 Department of Medicine, Surgery and Pharmacy, University of Sassari, 07100 Sassari, Italy

**Keywords:** breast reconstruction, breast hypertrophy, ptosis, post-bariatric, algorithm, mammoplasty, mastectomy

## Abstract

Immediate implant-based breast reconstruction in patients with large and ptotic breasts may be challenging due to skin redundancy. The use of a reduction mammoplasty pattern for the mastectomy skin excision has proven to be a reliable option for these patients as it allows for a better shape, projection, and symmetrization. This approach has been described in the literature for both one- and two-stage reconstruction with either sub- or pre-pectoral reconstruction with an acellular dermal matrix (ADM) or non-biological mesh. One-stage immediate breast reconstructions have a positive significant impact on patients’ psychosocial well-being and quality of life. The purpose of this paper is to describe an institutional algorithm that allows one to perform one-stage implant-based breast reconstructions in patients with large and ptotic breasts.

## 1. Introduction

It is a well-known fact that one-stage immediate breast reconstruction has a positive significant impact on patients’ psychosocial well-being and quality of life [1,2,3]. Nevertheless, immediate breast reconstruction in large and ptotic breasts can be challenging due to skin redundancy after skin-sparing mastectomy. The use of a traditional elliptical pattern usually flattens the breasts and leaves noticeable scars medially on the breast to avoid dog-ears. The Wise (Inverted T) [4,5] mammoplasty pattern skin excision, instead, can reduce the redundant skin envelope while lifting and restoring an appropriate shape of the breast. Moreover, this technique maximizes the outcome in terms of symmetrization, as reduction mammoplasty on the opposite site constitutes most of the time required. A major weakness of this technique is that skin flaps are often long and unreliable, with potential skin necrosis at the T junction and subsequent implant exposure [6,7,8]. Coverage of the inferior aspect of the implant with an inferiorly based dermal flap spared from the inferior aspect of the reduction pattern has been first proposed by Bostwick [9] to address the issue. In the original technique, a submuscular pocket was created by raising pectoralis major and serratus muscles, which were sutured to each other and to the dermal flap over a permanent implant; moreover, the nipple–areola complex (NAC) was moved as a full-thickness skin graft. Over the years, several variations have been described in the literature. As pre-pectoral reconstruction with the assistance of matrices was gaining popularity in the last decade [10], their application in breast reconstruction after Wise Pattern Mastectomy (WPM) has been advocated. Matrices can be divided into Acellular Dermal Matrices (ADMs) and synthetic meshes. ADMs are human-, bovine-, or porcine-derived engineered tissue grafts that work as a scaffold for the patient’s own cells and vessels to grow, thereby creating an extra tissue layer [11]. First mentioned as skin replacements in burn patients [12], their use in reconstructive surgery [13] has expanded over the years, as they proved to be reliable in a wide range of applications, from surgical oncology [13,14,15,16] to benign condition treatment [17,18,19]. Synthetic meshes are sheets knitted from permanent or absorbable fibers and have proven to be reliable in different surgical procedures, such as abdominal and pelvic surgery [20,21], where they are used to support organs and/or tissues, or in alloplastic breast reconstruction surgery [14,22], providing additional cover and support to the implant. The combination of matrices with the inferior dermal flap has been reported in the literature. Some authors [23,24] have described the use of matrices to cover the inferolateral aspect of the implant in a submuscular reconstruction; this provides a two-layer coverage of the inferior pole of the implant as the dermal flap is draped over the matrix. Others [25,26], instead, described their use in pre-pectoral reconstruction; matrices can be sutured to the superior border of the dermal flap and placed to cover the superior pole of the implant, or they can be used as a means for total implant coverage under the dermal flap. Different techniques have been also described to preserve the NAC, such as an inferiorly based dermal flap bearing the NAC [27] or a vertical bipedicle McKissock style flap [28]. Furthermore, an alternative reduction pattern for mastectomy skin excision has been proposed by Santanelli et al. [29] for patients requiring the skin excision of an upper quadrant due to the presence of a superficially located tumor or a scar from previous surgeries (e.g., lumpectomy, quadrantectomy) that may increase the risk of Wise skin flap necrosis. In case of unilateral reconstruction with concomitant contralateral reduction mammoplasty, Marongiu et al. [30] proposed the use of an autologous dermal patch obtained from the contralateral breast’s lower pole to complete the pre-pectoral pocket and better cover the implant.

One-stage immediate breast reconstruction has proven to be a highly acceptable treatment for patients requiring mastectomy. It is associated with a high quality of life, short reconstructive journey, and low procedure-related morbidity [1,2,3]. Several techniques have been proposed in the literature for immediate breast reconstruction in patients with macromastia and ptotic breast. Hence, the authors’ aim is to describe an institutional algorithm that allows one to perform one-stage, direct-to-implant breast reconstruction in patients with large and ptotic breasts.

## 2. Materials and Methods

A prospective application of the present algorithm was performed from October 2021 to the present time at the University Hospital Trust of Sassari, Italy. All the mastectomies were performed by Breast Surgeons of the Breast Unit, while immediate reconstructions were performed by Plastic Surgeons of the Plastic Surgery Unit.

We retrospectively analyzed the medical records of all the patients undergoing skin/nipple sparing Mastectomy with Wise pattern (type IV [31]) or modified Wise pattern (type V [29]), immediate breast reconstruction, and concomitant contralateral reduction mammoplasty, using the Algorithm from October 2021 to September 2022. Demographic, clinical preoperative, surgical, and postoperative information, such as body mass index (BMI), comorbidities, tobacco use, anthropomorphic measurement, type of reconstruction, implant type and volume, complications, and 3-month follow up evaluation, were recorded. Postoperative complications of interest were skin flap necrosis (either partial or full thickness, in relation to whether it was involving the dermal layer with subcutaneous tissue exposition), hematoma, seroma, surgical site infection, nipple necrosis, and implant loss. Early postoperative evaluation at three months of follow-up included both aesthetic and patient satisfaction outcome assessments. All the patients were asked to rate their satisfaction from “poor” to “excellent”. A third-party plastic surgeon of our unit was similarly asked to rate the aesthetic result from ‘‘poor’’ to ‘‘excellent’’.

### 2.1. Inclusion and Exclusion Criteria

The algorithm is applicable on female patients with large and ptotic beasts, including obese and post-bariatric patients. Inclusion criteria are nipple to sternal notch distance ≥ 26 cm and areola to inframammary fold distance ≥ 8 cm.

Exclusion criteria are history of previous radiotherapy, BMI > 40, heavy smoking habit (more than 20 cigarettes/day), uncontrolled diabetes, vasculitis, collagen diseases, or inflammatory breast cancer.

### 2.2. Algorithm

**Figure 1:**
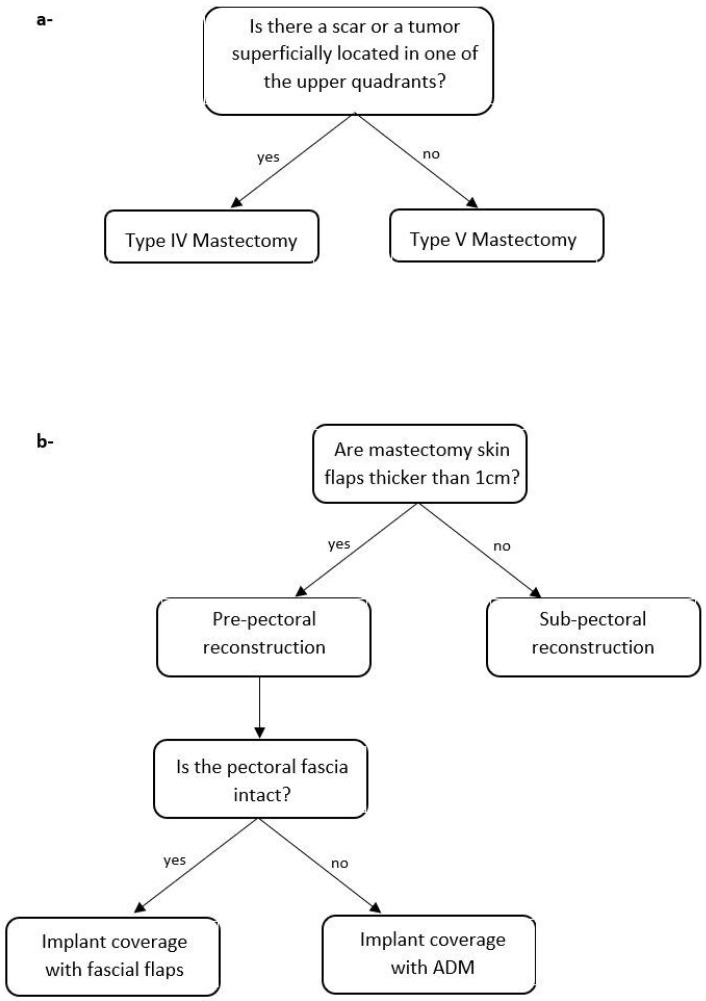
Institutional algorithm for immediate implant-based breast reconstruction in large and ptotic breasts: (**a**) preoperative evaluation; (**b**) intraoperative evaluation.

**Figure 2:**
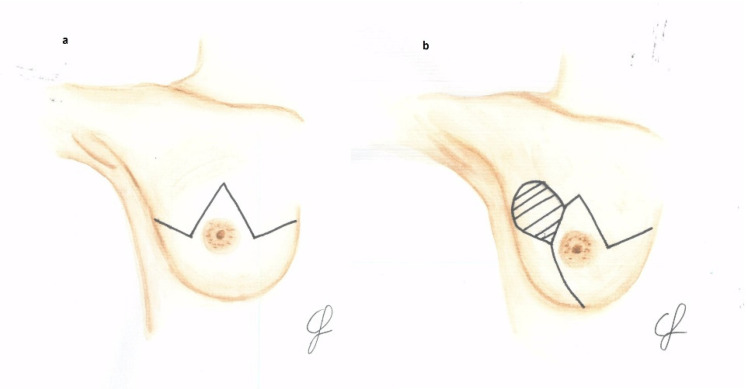
Preoperative markings: (**a**) Wise skin excision pattern; (**b**) modified-Wise skin excision pattern; dashed area shows the upper quadrant that has to be removed.

The first part of the algorithm (Figure 1a) demands a multidisciplinary preoperative evaluation of the tumor location, the corresponding need of skin resection to pursue oncological radicality, and the presence of a scar from previous surgeries (e.g., lumpectomy, quadrantectomy) in order to select the proper skin excision pattern for mastectomy. In case the breast tumor does not require skin resection, the Wise (Type IV [31]) reduction mammaplasty skin excision pattern is selected (Figure 2a). Alternatively, in case the tumor is superficially located in one of the upper quadrants, the modified Wise pattern (type V) described by Santanelli et al. [29] is chosen (Figure 2b); likewise, if a scar located in an upper quadrant may increase the risk of Wise skin flap necrosis, the latter pattern is preferred. Small variations of the preoperative drawing may be performed in the case of previous scars. In case of unilateral reconstruction, a contralateral Wise pattern reduction mammoplasty is planned.

**Figure 3:**
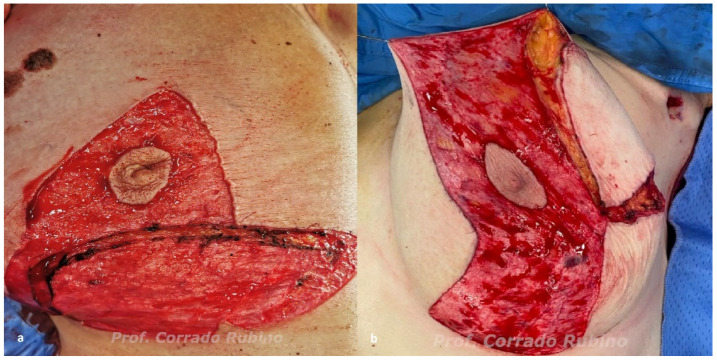
Different types of access for nipple/skin-sparing mastectomy: (**a**) type IV mastectomy is performed through a full thickness incision performed along the superior border of the inferior dermal flap; (**b**) type V mastectomy is performed through a full incision performed along the borders of the upper quadrant that has to be removed.

The reduction mammoplasty pattern and the inferior dermal flap are first de-epithelized. The skin-sparing mastectomy is then performed by the breast surgeon through a different access according to the pattern type (Figure 3). The nipple–areolar complex may or may not be preserved according to the result of the intraoperative retro-areolar frozen biopsy histopathological assessment. Nevertheless, if NAC viability, assessed either clinically or with indocyanine green fluorescence [32], is doubtful, the surgeon may decide to remove it.

The second part of the algorithm (Figure 1b), instead, requires an intraoperative assessment of mastectomy skin flaps thickness, which, in our institution, relies on clinical examination using palpation and a ruler. Furthermore, pectoral fascia integrity is assessed. These evaluations allow the surgeon to identify three main different approaches to breast reconstruction.

**Figure 4:**
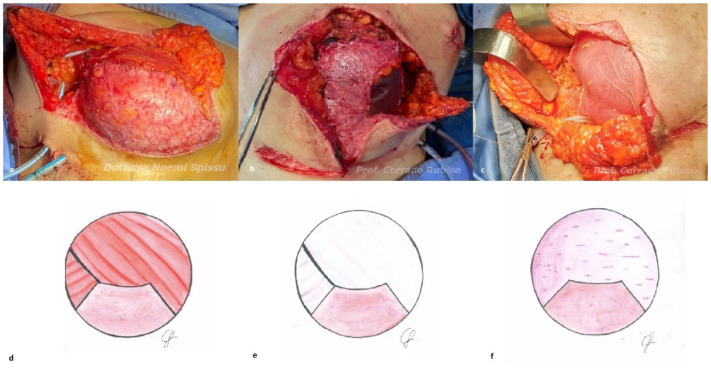
Intraoperative picture (**above**) and schematic drawing (**below**) of the three different options of implant coverage: (**a**,**d**) submuscular reconstruction; (**b**,**e**) pre-pectoral reconstruction and implant autologous coverage with fascial flaps; (**c**,**f**) pre-pectoral reconstruction with ADM.

Case 1—subpectoral reconstruction (Figure 4a,d): if the mastectomy skin flap’s thickness is ≤1 cm, a submuscular approach is performed. A pocket is first created by raising pectoralis major. The free edge of the muscle is then sutured to the upper border of the dermal flap, starting medially. When most of the pouch is closed, the implant is inserted, and the final suturing is performed. In case of big implants, the lateral aspect may need to be secured with the elevation of a serratus fascial flap, which is sutured to the dermal flap and the pectoralis major muscle.

Case 2—pre-pectoral reconstruction and implant autologous coverage with fascial flaps (Figure 4b,e): if the mastectomy skin flaps are thicker than 1 cm, a pre-pectoral reconstruction can be performed. In case the pectoral fascia is left intact after the mastectomy, the superior and lateral aspects of the implant can be covered by a pectoral and the serratus fascia flaps, respectively, as recently described by the authors of [33]. The pre-pectoral pocket is first created by harvesting a superiorly based fascial flap from the pectoralis major muscle and a laterally based fascial flap from the serratus muscle. The pouch is partially closed by suturing the superior edge of the dermal flap to the pectoral fascia and a permanent silicone implant is then placed into the pocket. Final suturing between the three flaps is performed in order to achieve complete autologous coverage of the implant.

Case 3—pre-pectoral reconstruction and implant coverage with ADM (Figure 4c,f): if the pectoral fascia is not intact after the mastectomy, a pre-pectoral reconstruction with the use of an ADM is preferred. A pre-shaped porcine ADM Braxon^®^ Fast (Decomed S.rl., Venezia, Italy) is used in our institution. The ADM is first wrapped around the implant, and its edges are sutured with interrupted absorbable sutures. The ADM containing the implant is then placed into the pocket and secured to the chest wall with superior, medial, and lateral interrupted absorbable sutures; an additional suture is sewed between the anterior sheet of the ADM and the subcutaneous tissue of the dermal flap and of the mastectomy skin flap in order to avoid any displacement and dead space.

### 2.3. Ethical Approval

The study was conducted in accordance with the Declaration of Helsinki (as revised in 2013). All the patients provided a written informed consent.

## 3. Results

From October 2021 to the present time, twelve patients were eligible for the described algorithm, which was used to select the proper skin excision pattern and type of breast reconstruction. Two patients underwent bilateral mastectomy, and ten patients underwent unilateral mastectomy with concomitant contralateral Wise pattern reduction mammoplasty for symmetrization. Mean patients age was 60 years (range 34–80 years) and mean BMI was 29.3 kg/m^2^ (range 24–37). Type V mastectomy was used in two patients (2 breasts); all the other cases were treated with a type IV mastectomy. Three patients underwent subpectoral reconstruction, six patients (eight breasts) underwent pre-pectoral reconstruction and implant coverage with fascial flaps, and three patients underwent pre-pectoral reconstruction with ADM. Mean implant volume was 390 cc (range 300–560 cc).

Complications included skin and/or NAC necrosis healed by secondary intention (14.3%) and that required surgical revision (28.6%), seroma (7.1%) hematoma (7.1%), and implant removal (7.1%). The most common complication was skin necrosis, which occurred in six breasts. One of these patients developed a seroma after the surgical revision and a subsequent periprosthetic infection, leading to implant removal.

**Figure 5:**
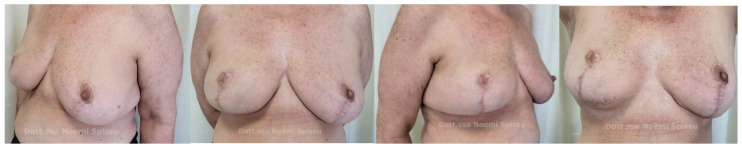
Clinical result at three months of follow-up. The patient was treated with type IV nipple-sparing mastectomy on the right side with submuscular reconstruction (460 cc implants) and concomitant contralateral reduction mammoplasty.

The evaluation at the three-month follow-up (Figure 5) showed good aesthetic and patient satisfaction outcomes. No sign of implant lateral migration has been detected in all patients. Six patients described their satisfaction with the result as “good”, three as “very good”, one as “excellent”, one as “scarce” and, lastly, the patient with implant reconstruction failure described it as “poor”. The aesthetic evaluation was reported as “excellent” in one case, “very good” in three patients, “good” in six patients, and “scarce” in one case. The aesthetic evaluation was not performed on the patients that lost the implant.

## 4. Discussion

As shown by our early results, the present algorithm is effective in treating patients with large and ptotic breasts as it allows the surgeon to perform both immediate breast reconstruction and concomitant symmetrization in one single procedure. This approach can, at the same time, address the oncological issue, as well as the one related to macromastia and/or ptosis, with a satisfactory aesthetic result and a positive impact on the psychosocial well-being of the patient. Complication rates are comparable to the ones presented in the literature [7,8,34,35]. One single implant loss occurred in our series. Pre-pectoral reconstruction with a 560 cc silicone implant covered with fascial flaps was selected for this case. The implant loss is likely to be related to the volume of the implant, which was probably too big.

Wise pattern mastectomy with an inferior dermal flap and submuscular direct-to-implant reconstruction has been well described in the literature [31,36,37]. We prefer to use this approach when mastectomy skin flap thickness is scarce. In these cases, skin viability is reduced with a high risk of skin necrosis; likewise, it is more likely to observe a visible implant rippling, making the subpectoral approach a better option. Mastectomy skin flaps thinner than 0.8 cm are, as a matter of fact, associated with a higher risk of ischemic complication, as recently described in the literature [38,39,40]. Despite this, we prefer to use a cut-off of 1 cm to ensure better implant coverage and to address the risk of visible rippling. The risk of rippling in pre-pectoral breast reconstruction has been associated [41] to a preoperative pinch test <2 cm. Hence, with mastectomy skin flaps thinner than 1 cm, we prefer a submuscular reconstruction to address both above-mentioned issues. Intraoperative use of indocyanine green fluorescence [32] may represent an additional tool to assess tissue perfusion. In our series, we were only able to use this method with two patients as the device was not available before. When mastectomy skin flaps are thicker than 1 cm, instead, we would rather perform a pre-pectoral reconstruction, as it is known to reduce postoperative pain with better functional outcomes [42,43]. In case the pectoral fascia is left intact after the mastectomy, the use of two fascial flaps [33] harvested from the pectoralis and serratus muscles is used to complete the pre-pectoral pocket. This technique provides complete autologous coverage of the implant; however, most importantly, it secures the superior and the lateral aspects of the implant, avoiding any lateral displacement. Moreover, in comparison to ADM or synthetic meshes [44,45], the use of fascial flaps for pre-pectoral reconstruction has a lower cost per procedure and no risk of phlogistic reaction. Recent findings show that pectoral fascia preservation during mastectomy seems oncologically safe [46]; nevertheless, the literature about the topic is scarce and many centers routinely perform fascial removal. For these reasons, we prefer to leave the choice to the breast surgeon. In case the pectoral fascia is not intact after the mastectomy, pre-pectoral reconstruction with the use of ADM is performed. The use of matrices in pre-pectoral breast reconstruction allows the surgeon to actively position the implant in an ideal location, with better control and definition of the inframammary fold. Moreover, ADMs are meant to modulate the wound-healing process and integrate into the host tissue with neovascularization and cell repopulation, guiding the formation of a soft and thin fibrous capsule around the implant [11]. As recently described in the literature [47], ADM-assisted alloplastic breast reconstruction has a significantly lower incidence of capsular contracture, even though further studies are needed to corroborate these findings.

According to our algorithm, the skin excision pattern is adaptable to a wide range of scenarios. The surgeon can select the proper reduction mammoplasty pattern in relation to the presence of scars from previous surgeries, the tumor location, and the desired final volume of the breast. In selected cases, J scar mammoplasty [48] or another reduction pattern may be used; likewise, different NAC pedicles may be selected according to the surgeon’s experience and preference. The same reduction patterns can also be used with autologous breast reconstruction, as already described in the literature [49,50].

The bilateral approach suggested by our algorithm can indeed achieve a better aesthetic and self-satisfaction outcomes. This approach allows, in one single procedure, to pursue a double goal: breast reconstruction and reduction mammoplasty. The use of a reduction mammoplasty pattern on both breasts can accomplish a better balance between the two sides, thus causing better aesthetic and patient self-satisfaction outcomes.

Patients with large and ptotic breasts undergoing this type of surgery must be selected carefully. Most of the time, these patients are obese, active smokers with different comorbidities; all these aspects are known to be associated with a higher risk of postoperative complications [51,52]. Likewise, the choice of a proper implant size is relevant, as bigger ones are associated with a higher risk of complications [53]. The authors prefer to use implants with a maximum volume of 500 cc. All these aspects demand preoperative counseling with the patient to address patient expectations and elucidate the risk of postoperative complication in order to select the proper surgical plan.

Limitations of this study include the limited cohort size and the short length of the follow-up period. The use of Breast-Q [54] and the Validated Aesthetic Scale [55] are routinely performed in our unit at one year of follow-up; therefore, a longer follow-up will allow us to provide the results obtained from the aesthetic and patient satisfaction outcomes based on validated questionnaires.

## 5. Conclusions

The present algorithm is a relevant tool for plastic surgeons aiming to treat patients with large and ptotic breasts who are eligible for implant breast reconstruction. It is a comprehensive algorithm addressing both breasts that provides consistent aesthetic results in a one-stage procedure, using different skin reduction patterns and implant coverage methods.

## Data Availability

The data used and/or analyzed during the current study are available from the corresponding author upon reasonable request.
